# Identifying endophenotypes of autism: a multivariate approach

**DOI:** 10.3389/fncom.2014.00060

**Published:** 2014-06-06

**Authors:** Fermín Segovia, Rosemary Holt, Michael Spencer, Juan M. Górriz, Javier Ramírez, Carlos G. Puntonet, Christophe Phillips, Lindsay Chura, Simon Baron-Cohen, John Suckling

**Affiliations:** ^1^Cyclotron Research Centre, University of LiègeLiège, Belgium; ^2^Department of Psychiatry, Autism Research Centre, University of CambridgeCambridge, UK; ^3^Department of Signal Theory, Networking and Communications, University of GranadaGranada, Spain; ^4^Department of Computer Architecture and Technology, University of GranadaGranada, Spain

**Keywords:** autism spectrum condition, MRI, support vector machine, searchlight, endophenotype

## Abstract

The existence of an endophenotype of autism spectrum condition (ASC) has been recently suggested by several commentators. It can be estimated by finding differences between controls and people with ASC that are also present when comparing controls and the unaffected siblings of ASC individuals. In this work, we used a multivariate methodology applied on magnetic resonance images to look for such differences. The proposed procedure consists of combining a searchlight approach and a support vector machine classifier to identify the differences between three groups of participants in pairwise comparisons: controls, people with ASC and their unaffected siblings. Then we compared those differences selecting spatially collocated as candidate endophenotypes of ASC.

## 1. Introduction

Autism Spectrum Condition (ASC) is a lifelong neurodevelopmental condition affecting approximately 1% of population (Baron-Cohen et al., 2009), that causes impairments of social communication alongside unusually repetitive behavior, narrow interests and resistance to change. Despite research efforts carried out over last few years, ASC is one of the more difficult conditions to characterize (Brambilla et al., 2003) and its aetiology is still largely unknown.

Historically postmortem studies have revealed neuroanatomic abnormalities (Bailey et al., 1998) associated with ASC that can also be analyzed by means of *in vivo* neuroimaging techniques. Neuroimaging technology is advancing at an impressive pace and is having a huge impact in both research and clinical environments. Magnetic Resonance Imaging (MRI) is a medical imaging technology that allows detailed visualization of the internal structures of the body. It has been widely used in the neurosciences, including in many studies of ASC. For example, in Sears et al. (1999) and Courchesne et al. (2001), the authors compared the size of several brain regions in controls and ASC participants from structural MRI. A longitudinal study focusing on the volume of the brainstem of controls and ASC participants was presented in Jou et al. (2013). Additionally, in Hashimoto et al. (1992) the brain volume and head circumference of participants with ASC were examined and compared with control individuals.

Recent studies suggest the existence of an endophenotype [a heritable biomarker associated with a pathology that individuals may have regardless of whether they have developed the pathology or not (Gottesman and Gould, 2003)] of ASC (Nydn et al., 2011; Spencer et al., 2012) identifiable through the analysis of unaffected relatives, typically parents or siblings of autistic individuals. In fact, according to (Constantino et al., 2010), siblings of individuals with ASC are at increased risk of developing the condition, with a prevalence estimated to be >20 times that of the general population.

Recently developed machine learning techniques provide an opportunity to analyse the differences between unaffected siblings of autistic individuals and controls with no family history of ASC. Differences co-located with phenotypic differences identified in comparisons of controls and ASC participants are candidate endophenotypes of ASC and may be attributed to effects at the level of the genome or epigenome that confer familial risk for the condition.

The analysis techniques used in neuroimaging are usually divided into two groups. On the one hand, univariate techniques perform a statistical analysis at each voxel separately and do not take into account relationships amongst distant voxels, although statistical inference of voxel clusters has improved sensitivity. Despite this disadvantage, univariate methods are widely used, in part because of the interpretability of results and access to the methodology through standard software packages such as Statistical Parametric Mapping (SPM) (Friston et al., 2011). In fact, univariate methods have been recently used to study the endophenotype of autism. For example in Dalton et al. (2007), the authors used a *t*-test to analyse functional Magnetic Resonance Imaging (fMRI) from controls and unaffected siblings of ASC individuals. Another univariate approach was used in Salmond et al. (2003) and Peterson et al. (2006). In these works, MRI data from ASC children were analyzed by means of Voxel Based Morphometry (VBM) (Ashburner and Friston, 2000). This approach allows investigation of focal differences in brain anatomy by performing a voxel-wise comparison of the local concentration or volume of gray matter between two or more groups of participants. Indeed, VBM has been widely used in the study of ASC and several meta-analyses have been conducted (Cauda et al., 2011; Radua et al., 2011; Via et al., 2011; Nickl-Jockschat et al., 2012). Whilst there appears to be a consistency in the reports of case-control differences in occipital, temporal, and parietal lobes as well as the precentral gyrus, generally the extant literature is characterized by a significant level of between-study variability. Such discrepancies between studies are variously ascribed to methodological differences, variations in the ages and IQs of participants, diagnostic criteria (Nickl-Jockschat et al., 2012) and, most frequently, to the heterogeneity of the signs and symptoms of ASC (Volkmar and Pauls, 2003). With these issues in mind, techniques that increase statistical sensitivity have added value in structural MRI studies of ASC and particularly in study designs for endophenotype discovery where effect sizes are potentially reduced by the introduction of a group (siblings) hypothesized to lie intermediate between cases and controls.

On the other hand, multivariate approaches, including machine learning, analyse each image as a whole and explicitly consider the inter-relationships across voxels. Here, effects due to brain structure or function as well as confounding and error effects are assessed statistically both at each voxel and as interactions among voxels (Friston et al., 2011). The main drawback to be addressed is the so-called small sample size problem (Duin, 2000), which occurs when the number of variables (voxels in case of neuroimaging) to be analyzed is significantly greater than the sample size (i.e., number of images) used in the study.

Searchlight analysis (or information mapping) is a recently described methodology based on multivariate pattern analysis (MVPA) that address the small sample size problem by dividing the brain volume into small regions of just a few voxels (Kriegeskorte et al., 2006; Kriegeskorte and Bandettini, 2007). It has been successfully used for classification problems [see for example (Illán et al., 2011)]. However, in this work it is used to highlight the locations where participants from two groups have differing patterns of brain tissue volume.

Here we demonstrate an original methodology to analyse structural MR images based on a multivariate exploration carried out with a searchlight approach. The primary goal is to discover evidence for the existence of an endophenotype of ASC. This condition has been intensively studied through univariate approaches, such as the ones mentioned above, and by directly measuring the size of several brain regions (Rojas et al., 2004; Webb et al., 2009). However, in our opinion, there is still room for the application of multivariate strategies that provide higher statistical sensitivity and allow corroborating or refuting the results reported in univariate studies. In addition, we hypothesized that multivariate approaches like the one proposed in this work provide a better way of analysing neuroimaging data by looking for differences between groups. Therefore, multivariate methods can realize differences even when univariate methods find no differences. Our hypothesis is based in the fact that multivariate approaches analyse the data not only at voxel level but also taking into account the relations between voxels. Thus, differences in those relations can be only found with multivariate methods.

We studied 132 whole-brain structural MRI from adolescents divided into three groups: ASC participants, unaffected siblings of ASC participants, and individuals with no family history of ASC (Table 1), referred to hereafter as “ASC,” “siblings,” and “controls” respectively. After normalizing the MRI volumes to a standard stereotactic space, we performed a searchlight analysis comparing “ASC” vs. “controls” and “siblings” vs. “controls.” The resulting information maps identified overlapping regions of between-group difference that may be considered as elements of an endophenotype of ASC. It is worth noting that this cohort was previously analyzed with VBM and no significant differences were found.

**Table 1:** **Demographic details of participants**.

**Number**		**Sex**		**Age**			**IQ**		
		**M**	**F**	*μ*	*σ*	**range**	*μ*	*σ*	**range**
ASC	52	35	17	14.49	1.74	12–18	104.6	15.89	73–146
Siblings	40	12	28	14.86	2.12	12–18	113.1	10.06	88–133
Controls	40	20	20	15.06	1.61	12–18	112.4	11.12	83–136

μ and σ are the average and the standard deviation, respectively.

## 2. Materials and methods

### 2.1. Study description

The study cohort comprised 132 participants aged between 12 and 18 years. Fifty-two (52) had a diagnosis of ASC, either classic autism or Asperger syndrome, 40 participants were unaffected siblings of individuals with ASC. Forty (40) controls with no family history of ASC were also recruited. Demographic details are gathered in Table 1.

All ASC participants met *Diagnostic and Statistical Manual of Mental Disorders*, fourth edition criteria (American Psychiatric Association et al., 1994) for autism or Asperger syndrome and were positive on the Autism Diagnostic Interview-Revised (Lord et al., 1994) and the ADOS-G (Losh et al., 2009). Further details on recruitment are given in references (Spencer et al., 2011, 2012).

The study was given ethical approval by the Cambridgeshire 1 Research Ethics Committee, and all participants and their parents provided written informed consent.

Data collection took place at the Medical Research Council, Cognition and Brain sciences unit, on a Siemens Tim Trio (Siemens Medical Solutions, AG, Erlangen, Germany) operating at 3T. High-resolution T1-weighted three-dimensional magnetization-prepared rapid acquisition gradient-echo (MP-RAGE) structural images were acquired with the following parameters: slice thickness = 1 mm; *TR* = 2300 ms; *TE* = 2.98 ms; field of view = 256 × 240 × 176 mm; flip angle = 9°; voxel size = 1 × 1 × 1 mm.

All MRI datasets were first segmented into their component tissues (gray and white matter) and then normalized to a standard stereotactic space of the Montreal Neurological Institute (MNI) using SPM software (Friston et al., 2011). Specifically, we used the Diffeomorphic Anatomical Registration Through Exponentiated Lie Algebra (DARTEL) (Ashburner, 2007). This algorithm normalizes both segmented gray and white matter images from all participants in an integrated, iterative procedure by computing a flow field which can then be expressed as both forward and backward image deformations. The MNI-normalized gray matter volume maps were used in the subsequent analyses.

### 2.2. Multivariate analysis based on machine learning

During the last decade, many research efforts have been focused on MVPA as a promising way of analysing high dimensional data. The growth of multivariate approaches is partly due to the recent advances in machine learning which provide more accurate statistical classifiers with the benefits of generalization. In that sense, support vector machines (SVM) have recently attracted the attention of the pattern recognition community because of the merits derived from statistical learning theory (Vapnik, 1995, 1998) developed by Vladimir Vapnik in late 1990s.

A SVM classifier builds a function *f*: *R*^*N*^ → {±1} using the training data (*N*-dimensional patterns **x**_*i*_ and their class labels *y*_*i*_) so that *f* is able to predict the label *y* of a new example **x**. The computation of function *f* is based on the calculation of a hyperplane, called the maximal margin hyperplane, that has the largest distance to the closest training data point of any class. Then, this hyperplane is used to classify new data points of unknown class. Formally, the decision hyperplane is defined as:

(1) $$g(x)=w^{T}x+w_{0}=0,$$

where **w** is the weight vector, orthogonal to the decision hyperplane, and *w*_0_ is the threshold. When no linear separation of the training data is possible, SVM can work effectively in combination with kernel techniques so that the hyperplane defining the SVM corresponds to a non-linear decision boundary in the input space (Müller et al., 2001).

SVM has been successfully used in a number of problems in different fields and is one of the most prevalent classifiers in neuroimaging-based classification tasks (Shen and Ji, 2009; Swiderski et al., 2009; Cuingnet et al., 2011; Zhang et al., 2011), such as the development of computer aided diagnosis systems for neurodegenerative disorders (López et al., 2009; Segovia et al., 2010, 2012). As with other statistical classifiers, SVM may be also used to highlight the differences between two groups of images by means of a searchlight approach. Searchlight (Kriegeskorte et al., 2006) divides the brain volumes into small regions that are independently analyzed. All the regions are of equal size and shape and cover the entire parenchyma of the brain. They are defined by following a systematic procedure with no prior knowledge about the groups that are being compared. As a result of applying this technique, a map is obtained derived from the discriminating power of different brain regions.

In this work, we utilized a searchlight approach and support vector machine classification to look for anatomical differences between controls and ASC participants, and between controls and unaffected siblings of ASC participants. Then, by comparing both maps of classification accuracies we estimated potential endophenotypes of ASC. Thus, the exploration implemented here makes two comparisons of two groups and creates a map that assigns a discrimination power to each voxel. However, the analysis is not carried out in a univariate way (voxel by voxel), but it is performed over small regions and then the result obtained for a region is assigned to all the voxels in that region. We defined as many regions as voxels resulting in a high rate of overlapping and allowing for exhaustive exploration of the image space. Cubic regions with a side of 5 voxels were used, leading to regions containing 125 voxels.

For each region we used a *k*-fold cross-validation (CV) scheme along with a SVM classifier to estimate the accuracy of classifying the images in the two groups. A high accuracy rate indicates that the analyzed region contains large differences between the two groups. On the other hand, a low accuracy rate, about 50%, suggests only small differences. The classification procedure used a linear kernel along with the SVM classifier (with parameter *C* fixed to the commonly accepted value of *C* = 1) and estimated the accuracy rates by means of CV, which is an effective method for estimating the risk of a classifier (Cortes and Vapnik, 1995; Müller et al., 2001). Specifically, we used a 10-fold scheme that provides similar estimation errors than leave-one-out (Varma and Simon, 2006) with a smaller computational load (an important issue to be taken into account in our experiments since we performed many thousands of CV loops).

Subsequently, the significance of the accuracy obtained with each region was assessed with a non-parametric test. This additional test consisted of repeating 1000 times the same classification procedure but using random labels. In this way we were able to estimate the probability that an accuracy rate was obtained by chance (Good, 2000). A *p*-value was then computed as the number of repeats where the accuracy obtained with the random labels was larger than that obtained with the true labels, divided by 1000. In order to address the multiple comparison problem (Miller, 1966), a false discovery rate control based on the Benjamini-Hochberg procedure (Benjamini and Hochberg, 1995) was applied. Accordingly, the tests with high *p*-values (i.e., *p*-values higher than an *ad-hoc* threshold related to the number of simultaneous tests) were discarded.

Finally, we assigned to each voxel the highest accuracy rate obtained for all the regions that *included* that voxel. The pseudocode for the searchlight procedure is shown in Algorithm 1.

**Algorithm 1:**
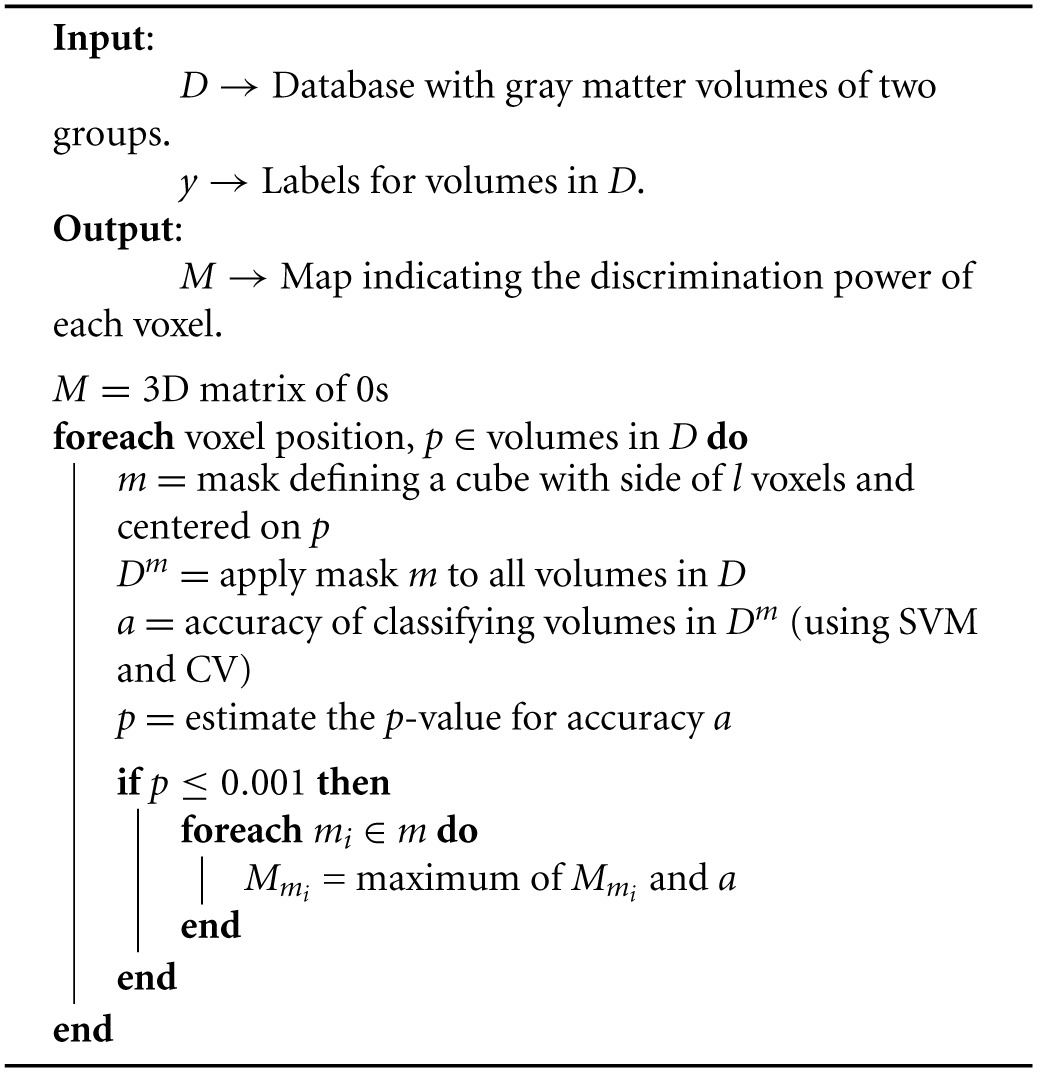
**Implementation of the SVM-based searchlight approach**.

## 3. Experiments and results

**Figure 1:**
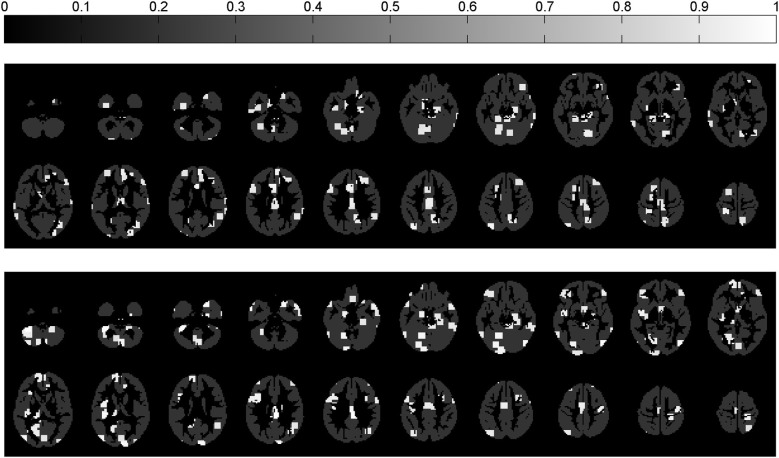
**Location of the significant regions (estimated through the Benjamini-Hochberg procedure with α = 0.05) when classifying controls vs. ASC (top) and controls vs. siblings (bottom)**. The intensity of the white color indicates the accuracy achieved for that region. The accuracy rate varies from 65.22 to 77.17% for the first classification and from 66.25 to 82.50% for the second one.

**Figure 2:**
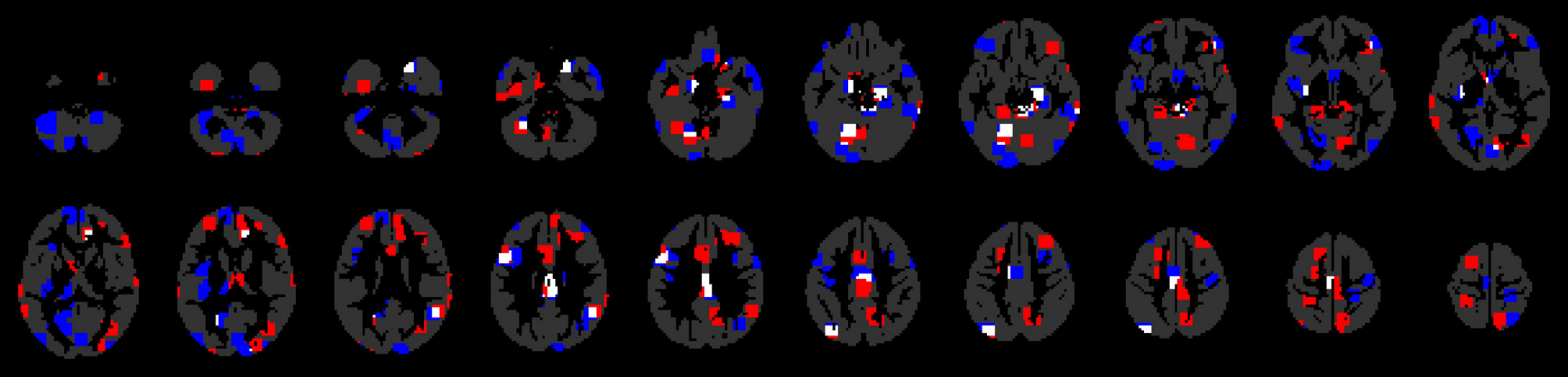
**Comparison of between-group difference maps shown in Figure 1**. Red: significant regions for the controls vs. ASC participant's classification; blue: significant regions for the controls vs. siblings classification; white: the common regions for both classifications.

The experiments were performed in three steps: First, we estimated the locations with different patterns in controls and ASC participants using the multivariate approach described in section 2.2. Secondly, we followed the same procedure to differentiate between controls and unaffected siblings of ASC participants. Finally, we estimated the similarities between the two maps computed in previous steps by finding the regions of significant difference common for both. The result of the first two steps is shown in Figure 1 and the final comparison (step 3) is shown in Figure 2. In order to analyse the locations of the regions highlighted by the algorithm, we calculated the brain regions involved by means of the Anatomical Labeling Atlas (AAL) (Tzourio-Mazoyer et al., 2002). Table 2 shows the structural regions containing areas highlighted in Figure 1 and the percentage of brain region they cover. Regions covered by a relatively large area in both classifications (shown in the last column) are candidate endophenotypes of ASC. This analysis facilitates the comparison of results obtained in this work with the extant literature.

**Table 2:** **Size of the significant regions for classifications of control vs. ASC (second column) and controls vs. siblings (third column), grouped by anatomical region**.

**Anatomical region (hemisphere)**	**Controls vs ASC (%)**.	**Controls vs siblings (%)**.	**Common area (%)**
Cerebelum (left)	394 (9.71)	821 (20.24)	146 (3.60)
Parietal lobule (right)	123 (9.33)	162 (12.28)	100 (7.58)
Occipital (left)	239 (11.86)	289 (14.34)	74 (3.67)
Angular gyrus (left)	75 (18.25)	141 (34.31)	71 (17.27)
Inferior frontal gyrus (left)	89 (8.64)	265 (25.73)	67 (6.50)
Temporal gyrus (right)	426 (10.71)	377 (9.48)	62 (1.56)
Cingulate gyrus (left)	152 (11.27)	197 (14.60)	59 (4.37)
Parietal lobule (left)	111 (6.95)	79 (4.94)	41 (2.57)
Parahippocampal gyrus (right)	51 (13.35)	75 (19.63)	31 (8.12)
Hippocampus (left)	36 (11.92)	89 (29.47)	30 (9.93)
Thalamus (right)	64 (19.22)	38 (11.41)	27 (8.11)
Cerebelum (right)	262 (6.05)	273 (6.31)	24 (0.55)
Vermis	192 (26.82)	44 (6.15)	24 (3.35)
Cingulate gyrus (right)	394 (29.76)	62 (4.68)	23 (1.74)
Area triangularis (left)	101 (10.64)	91 (9.59)	21 (2.21)
Precuneus (right)	296 (24.79)	74 (6.20)	18 (1.51)
Calcarine sulcus (left)	31 (3.54)	178 (20.32)	17 (1.94)
Precuneus (left)	33 (2.52)	230 (17.60)	15 (1.15)
Putamen (left)	21 (5.98)	79 (22.51)	15 (4.27)
Thalamus (left)	65 (18.90)	139 (40.41)	11 (3.20)
Globus pallidus (left)	38 (43.18)	11 (12.50)	8 (9.09)
Middle temporal pole (right)	43 (9.35)	91 (19.78)	7 (1.52)
Superior frontal gyrus (right)	120 (3.94)	146 (4.79)	7 (0.23)
Supplementary motor area (left)	24 (3.38)	22 (3.09)	7 (0.98)
Amygdala (left)	8 (10.53)	6 (7.89)	6 (7.89)
Inferior frontal gyrus (right)	125 (10.48)	160 (13.41)	6 (0.50)
Fusiform gyrus (right)	10 (1.12)	65 (7.30)	5 (0.56)
Middle frontal gyrus (left)	142 (6.59)	252 (11.69)	5 (0.23)
Calcarine sulcus (right)	109 (15.55)	74 (10.56)	4 (0.57)
Caudate nucleus (left)	42 (11.54)	5 (1.37)	4 (1.10)
Lingual gyrus (right)	139 (16.22)	32 (3.73)	4 (0.47)
Angular gyrus (right)	13 (2.02)	22 (3.42)	3 (0.47)
Parahippocampal gyrus (left)	34 (10.12)	38 (11.31)	2 (0.60)
Superior temporal pole (right)	63 (11.71)	5 (0.93)	1 (0.19)
Area triangularis (right)	89 (10.29)	23 (2.66)	–
Caudate nucleus (right)	1 (0.27)	42 (11.29)	–
Cuneus (left)	–	23 (4.09)	–
Cuneus (right)	2 (0.40)	71 (14.12)	–
Fusiform gyrus (left)	72 (9.13)	48 (6.08)	–
Gyrus rectus (left)	–	5 (1.51)	–
Gyrus rectus (right)	–	1 (0.37)	–
Hippocampus (right)	3 (0.94)	16 (5.02)	–
Insula (left)	–	230 (33.82)	–
Insula (right)	7 (1.07)	–	–
Lingual gyrus (left)	10 (1.27)	343 (43.69)	–
Middle frontal gyrus (right)	130 (5.66)	167 (7.27)	–
Middle temporal pole (left)	–	62 (21.23)	–
Occipital (right)	21 (1.30)	220 (13.58)	–
Olfactory cortex (left)	–	6 (6.00)	–
Olfactory cortex (right)	–	11 (9.48)	–
Paracentral lobule (left)	9 (1.96)	67 (14.60)	–
Paracentral lobule (right)	2 (0.70)	4 (1.40)	–
Postcentral gyrus (left)	48 (3.63)	81 (6.12)	–
Postcentral gyrus (right)	–	35 (2.55)	–
Precentral gyrus (left)	8 (0.71)	17 (1.51)	–
Precentral gyrus (right)	–	124 (11.10)	–
Rolandic operculum (left)	–	52 (15.57)	–
Rolandic operculum (right)	10 (2.16)	–	–
Superior frontal gyrus (left)	161 (5.09)	398 (12.57)	–
Superior temporal pole (left)	–	12 (2.43)	–
Supplementary motor area (right)	114 (14.21)	1 (0.12)	–
Supramarginal gyrus (right)	10 (1.48)	–	–
Temporal gyrus (left)	256 (6.99)	205 (5.60)	–
Transverse temporal gyri (left)	–	24 (28.92)	–
Transverse temporal gyri (right)	1 (1.16)	–	–

The last column (fourth) contains the size of the common area for both classifications. The size is given in mm^3^ and as the percentage of the total volume of the anatomical region.

**Figure 3:**
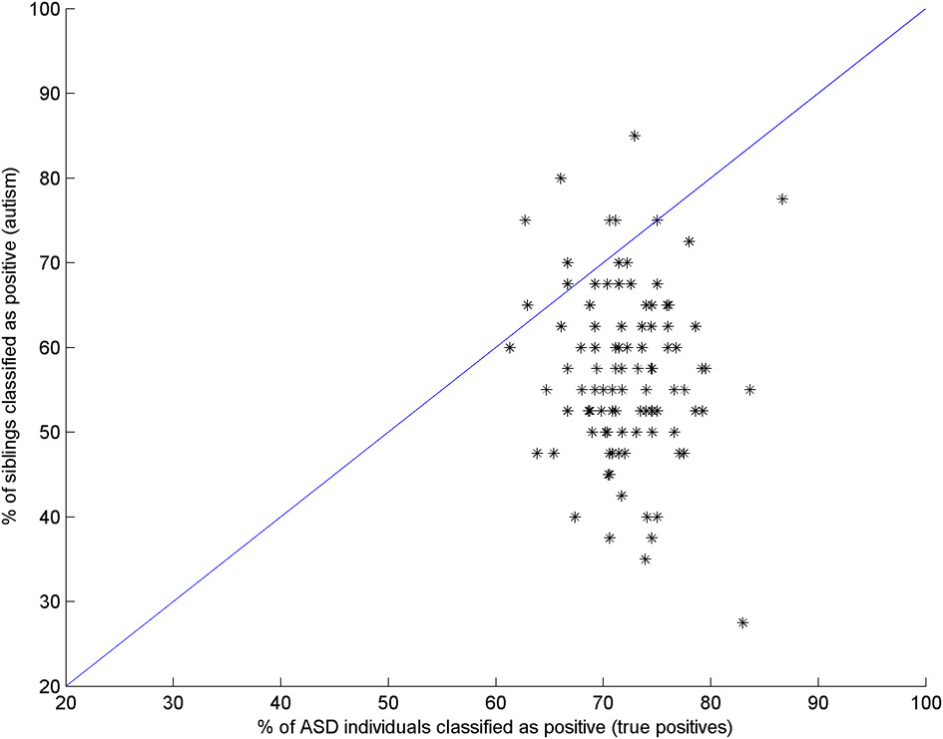
**Percentage of ASC individuals correctly classified as ASC by a classifier trained with controls and ASC data (abscissa) vs. the percentage of siblings classified as ASC by the same trained classifier (ordinate axis)**. Regions near to the blue line provide approximately the same accuracy in both classifications. This suggests they have similar patterns and corroborates the suggestion that they are endophenotypes of ASC.

In order to test if regions marked as common for both classifications indeed have the same pattern, an additional experiment was performed. For each significant region in the first classification, a SVM classifier was trained using controls and ASC participants, and the positive rate (percentage of ASC correctly classified) was then estimated through CV. Subsequently, the sibling data was evaluated with that trained classifier. For about 20% of the regions, the number of ASC individuals correctly classified as such was approximately the same (equal or in the 10% range) as the number of siblings classified as ASC. This reveals the existence of a common pattern for ASC and siblings in those regions and strengthens the suggestion that they are endophenotypes of ASC. Figure 3 plots the positive rate vs. the percentage of siblings classified as ASC. Note that 20% of regions mentioned above are located close to the blue line.

**Figure 4:**
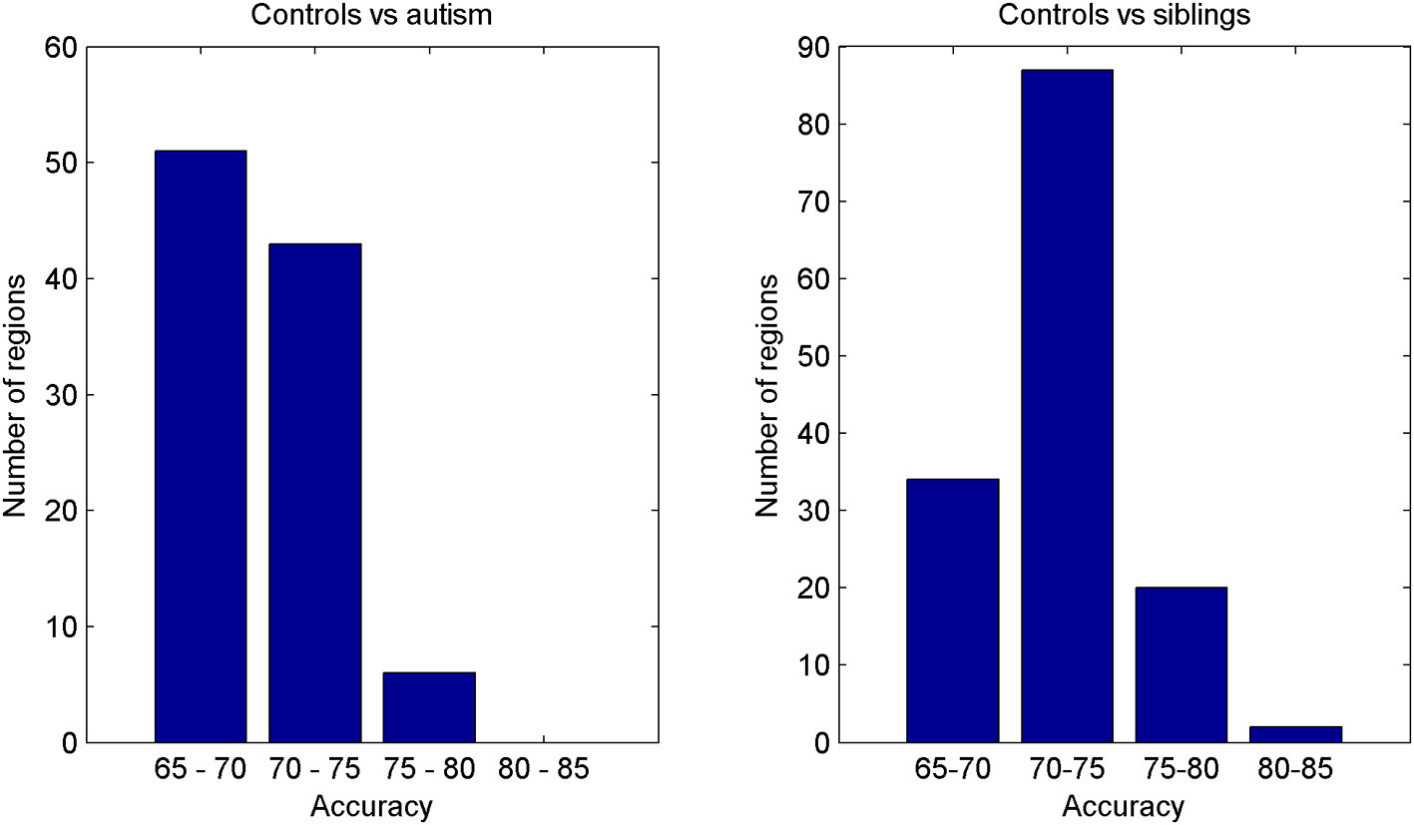
**Histograms of the number of regions for which the SVM classifier achieved significant accuracy rates when classifying controls vs. ASC participants (left) and controls vs. siblings of ASC participants (right)**.

Regardless of the regional analysis it is reasonable to expect that classification of controls vs. ASC participants would achieve higher accuracy rates than classification of controls vs. siblings of ASC participants. After all, the siblings have not developed the condition and should be more similar to controls and thus separating these groups should be expected to be relatively more challenging. However, our experiments indicate the converse. Figure 4 shows the histograms with the number of regions for which the two classifications (controls vs. ASC and controls vs. siblings) achieved significant accuracies. Note the large differences in the intervals of 70–75 and 75–80% of accuracy.

## 4. Discussion

The results presented above demonstrate that structural differences exist in gray matter maps derived from neuroimaging data comparing controls and ASC participants, as well as between controls and unaffected siblings of ASC individuals. In the first case the regions containing differences are mainly located in temporal gyrus, cerebellum, right cingulate gyrus, right precuneus, left occipital and vermis, whereas differences for the second comparison are mostly located in cerebellum, left frontal gyrus, right temporal gyrus, left lingual gyrus, left occipital, and insula (a complete list of the regions is shown in Table 2). A large number of common regions are located in the cerebellum, the importance of which has been emphasized in previous studies of ASC (Salmond et al., 2003). Specifically, left cerebellum was suggested as a neuroendophenotype for ASC in Peterson et al. (2006), where unaffected parents of autistic participants and controls were compared. These authors found abnormalities in the left cerebellar volume of the parents indicating the existence of similarities in altered brain structures of autistic individuals and their unaffected relatives.

The vermis is also a candidate region as a neuroendophenotype of ASC (group differences in both ASC compared to controls and siblings compared to controls covering 23 mm^3^ were found in this region). Smaller vermis volume in ASC participants than in controls was found in Kilman et al. (1997) and, more recently in Webb et al. (2009). Figure 2 and Table 2 show that differences in this region are also extant in the comparison between controls and siblings of ASC participants discovered in the present study.

Other previous works that looked for neuroendophenotypes of ASC reported that the amygdala was smaller in adults with ASC relative to the unaffected parents of children with ASC and age-matched controls (Rojas et al., 2004). In our analysis we found small differences in the left amygdala when comparing ASC participants vs. controls and siblings vs. controls. In addition the regions highlighted in both comparisons are of similar size. These results concur with those described in Dalton et al. (2007), where the authors compared adolescents diagnosed with ASC, unaffected siblings of ASC individuals and participants with no personal or familial history of ASC. They found that amygdala volume of ASC participants was similar to their unaffected siblings and significantly smaller when compared with a control group, which may have implications for social interaction and communication difficulties in autism and potentially subtle traits in siblings.

In general, it worth noting that an important part of the regions highlighted in our analysis and enumerated in Table 2 also appear relevant to differentiating between control and ASC individuals in a number of univariate studies (Cauda et al., 2011; Radua et al., 2011; Via et al., 2011; Nickl-Jockschat et al., 2012).

The results presented also suggest that our initial hypothesis on the enhanced ability of multivariate approaches to finding differences between groups of neuroimaging data is substantiated. The proposed method found significant differences in a dataset in which VBM had previously found no significant differences at levels of significance controlling for multiple comparisons. Furthermore, the locations of the observed differences match those reported by univariate studies using other, larger datasets.

Finally, some considerations about the size and shape of the regions, and the manner of exploration associated with the searchlight approach can be drawn. The original searchlight approach proposed by Kriegeskorte et al. (2006) used spherical regions. However, here we used cubes for computational simplicity, reducing the computation time of the procedure without producing adverse effects. In fact, a cube-based searchlight approach was successfully used in Illán et al. (2011) for classification purposes. In general, the size of the regions is a key control parameter for this approach since it controls the trade-off between localization and globalization. If the regions are small, the results (highlighted regions) will be more accurate with enhanced spatial localization, but the approach tends to the univariate approach with its attendant disadvantages. In addition, using smaller regions may result in an increase of the computational burden if region overlapping is limited. Conversely, if the regions are large, finding small areas of interest is more difficult or, if found, will be included in regions much larger than the area of interest, decreasing the localization power. As described in section 3, we used regions of 125 voxels that, in our opinion, represent a good trade-off between accuracy and computational burden. Furthermore, the exploration of the brain space may be undertaken with varying amounts of overlap of the regions. Clearly, overlapping allows a more exhaustive exploration and is particularly important when regions are large.

## 5. Conclusions

We have presented an original analysis of MRI images by means of a multivariate approach in order to identify candidate neuroendophenotypes of ASC. To this end, we first looked for differences between controls and ASC participants, and then compared these differences to those resulting from comparing controls and unaffected siblings of ASC participants.

The main novelty presented in this study is the algorithm used in the discovery of those differences: a multivariate approach that consists of performing an exhaustive examination of the brain space by means of a searchlight methodology combined with a support vector machine classifier.

The results reported here indicate that differences in cerebellum, parietal lobule, left occipital, left angular gyrus and, to a lesser extent, other regions listed in Table 2 can be considered neuroendophenotypes of ASC. Additionally, we corroborated the existence of separate, significant differences between controls and siblings of ASC participants that have not developed the condition. These findings, if replicated by other studies, may go some way to explaining the increased likelihood of unaffected siblings of ASC individuals developing the condition in later life.

### Conflict of interest statement

The authors declare that the research was conducted in the absence of any commercial or financial relationships that could be construed as a potential conflict of interest.
